# Supplementary material for: High-performance particulate matter including nanoscale particle removal by a self-powered air filter
Source: Nat Commun. 2020 Apr 3;11:1653. doi: 10.1038/s41467-020-15502-7 (PMC7125120; doi:10.1038/s41467-020-15502-7)
Supplement: Supplementary file 1 — Supplementary Information [file 41467_2020_15502_MOESM1_ESM.pdf]

Supplementary Information: **High-Performance Particulate Matter Including Nanoscale Particle Removal by a Self-powered Air Filter**

Guo-Hao Zhang, Qiu-Hong Zhu, Lei Zhang, Fang Yong, Zhang Zhang, Shuang-Long Wang, You Wang, Ling He* & Guo-Hong Tao*

College of Chemistry, Sichuan University, Chengdu 610064, China. (email: lhe@scu.edu.cn; taogh@scu.edu.cn)

This PDF file includes:

Supplementary Tables 1

Supplementary Figures 1 to 17

Supplementary References

Supplementary **Table 1 | Water, CH_2I_2 contact angle and surface free energy (γ^{tot}) of the ILP composites with different polymers (PAM, PVA and PVP) and amounts of ILs.** The γ^{tot} values were calculated by Owens two-zone mechanism of adhesion[1].

Film material	Water contact angle (°)	CH_2I_2 contact angle (°)	Polar surface energy γ^p (mN m^{-1})	Dispersive surface energy γ^d (mN m^{-1})	Total surface free energy γ^{tot} (mN m^{-1})
PAM	72.0	69.4	16.5	16.2	32.6
PVA	82.3	63.3	22.0	7.3	29.4
PVP	81.4	40.1	36.2	3.7	39.9
[C_4mim][OAc]-PAM (0.2)	55.4	64.3	16.5	28.4	45.0
[C_4mim][OAc]-PAM (0.5)	51.2	69.4	13.3	34.9	48.2
[C_4mim][OAc]-PAM (1)	48.4	59.9	17.9	32.6	50.5
[C_4mim][OAc]-PVA (0.2)	89.0	49.6	32.4	2.1	34.6
[C_4mim][OAc]-PVA (0.5)	76.5	52.2	27.7	8.1	35.8
[C_4mim][OAc]-PVA (1)	64.9	57.1	22.3	17.4	39.7
[C_4mim][OAc]-PVP (0.2)	65.6	41.6	31.4	12.5	43.9
[C_4mim][OAc]-PVP (0.5)	50.9	45.1	26.5	24.5	50.9
[C_4mim][OAc]-PVP (1)	40.4	43.8	25.4	32.3	57.7

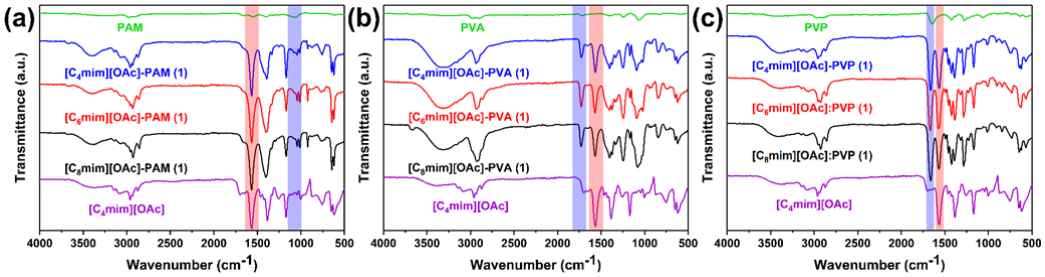

Supplementary **Figure 1 | FTIR spectra of different ILP composites.** (**a**) ILP composites with different ILs ([C_4mim][OAc], [C_6mim][OAc] and [C_8mim][OAc]) and PAM. (**b**) ILP composites with different ILs and PVA. (**c**) ILP composites with different ILs and PVP. Source data are provided as a Source Data file.

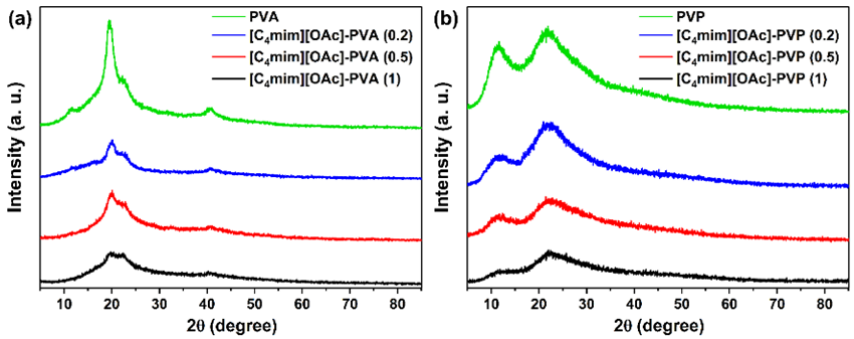

Supplementary **Figure 2 | PXRD patterns of different ILP composites.** (**a**) The ILP composites consisting of [C_4mim][OAc] and PVA. (**b**) The ILP composites consisting of [C_4mim][OAc] and PVP. Source data are provided as a Source Data file.

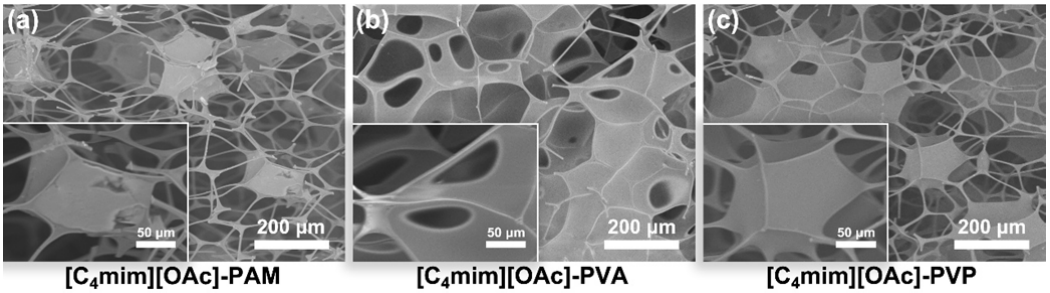

Supplementary **Figure 3 | SEM images of ILP@MF filters with different species of polymers.** (**a**) [C_4mim][OAc]-PAM. (**b**) [C_4mim][OAc]-PVA. (**c**) [C_4mim][OAc]-PVP.

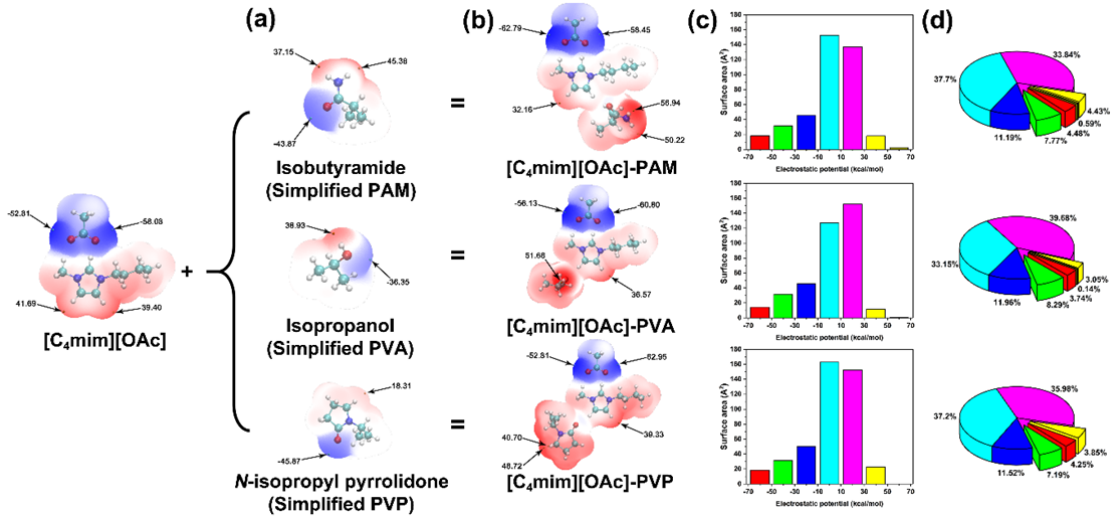

Supplementary **Figure 4 | ESP-mapped molecular vdW surface of the [C$_4$mim][OAc].** (**a**) simplified polymers. (**b**) ILP composites consisting of [C$_4$mim][OAc] and different polymers. (**c**) Surface area in each ESP range on the vdW surface of the ILP composites. (**d**) Area percent in each ESP range on the vdW surface. Source data are provided as a Source Data file.

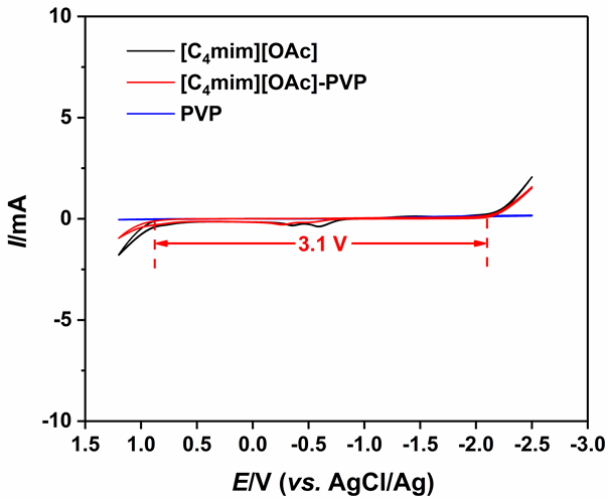

Supplementary **Figure 5 | Cyclic voltammograms of [C$_4$mim][OAc], PVP and [C$_4$mim][OAc]-PVP composite.** T = 298 K, v = 0.1 V s^{-1}. Source data are provided as a Source Data file.

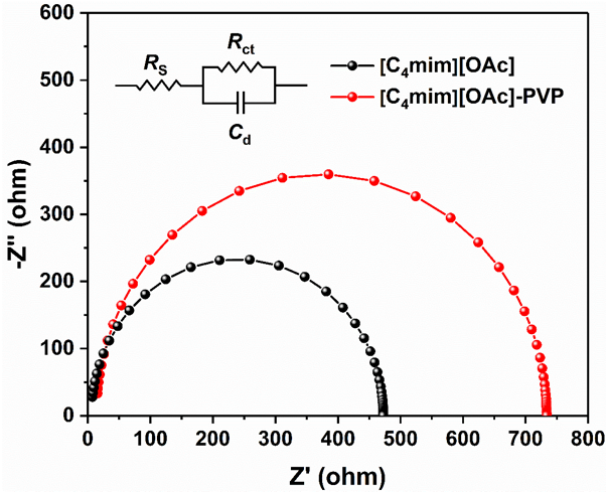

Supplementary **Figure 6 | The representative Nyquist plots of pure [C4mim][OAc] and [C4mim][OAc]-PVP composite.** The inset image is the equivalent circuit used for fitting the Nyquist plots. Source data are provided as a Source Data file.

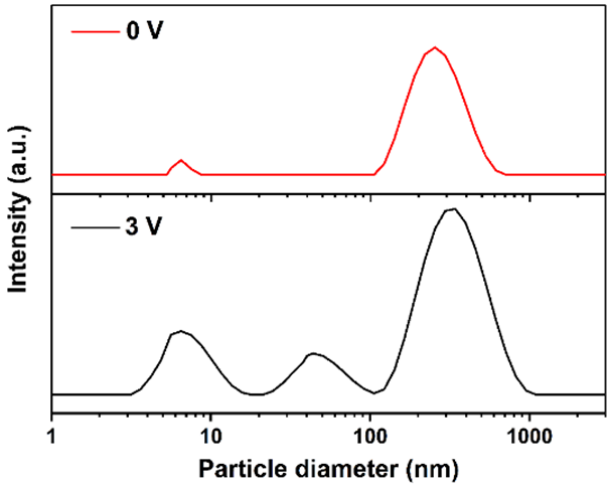

Supplementary **Figure 7 | DLS curves of the particles washed from the used [C4mim][OAc]-PVP@MF filter under different voltage.** Source data are provided as a Source Data file.

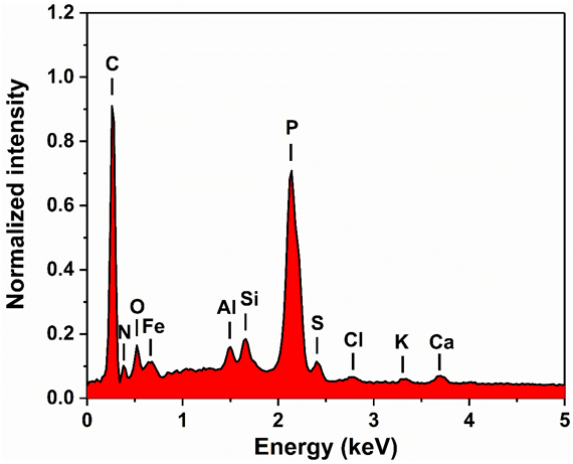

Supplementary **Figure 8 | The EDS spectrum of the charged [C_4mim][OAc]-PVP@MF filter after the filtration test.** Source data are provided as a Source Data file.

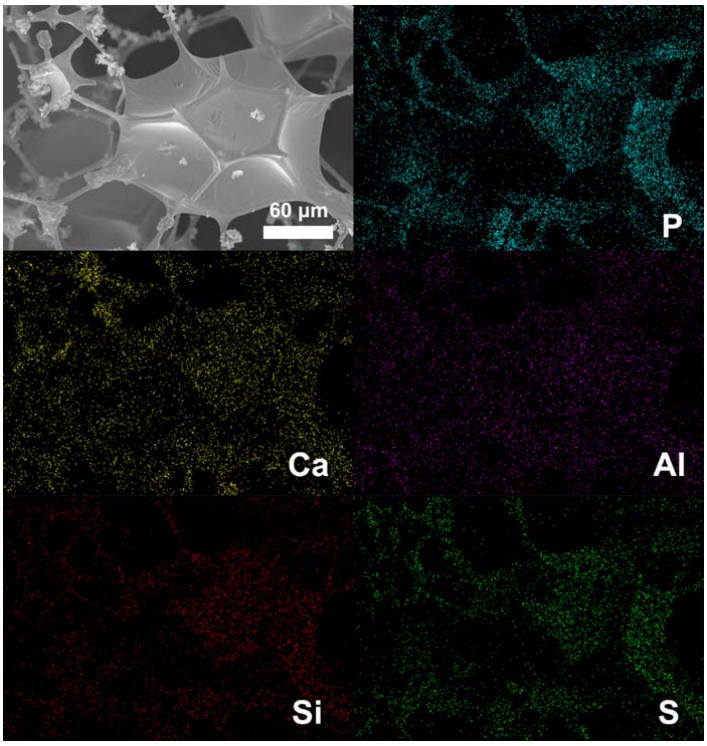

Supplementary **Figure. 9 | EDS mapping images of the charged [C_4mim][OAc]-PVP@MF filter after the filtration test.** Phosphorus (P; blue dots), calcium (Ca; yellow dots), aluminium (Al; purple dots), silicon (Si; red dots), and sulfur (S; green dots).

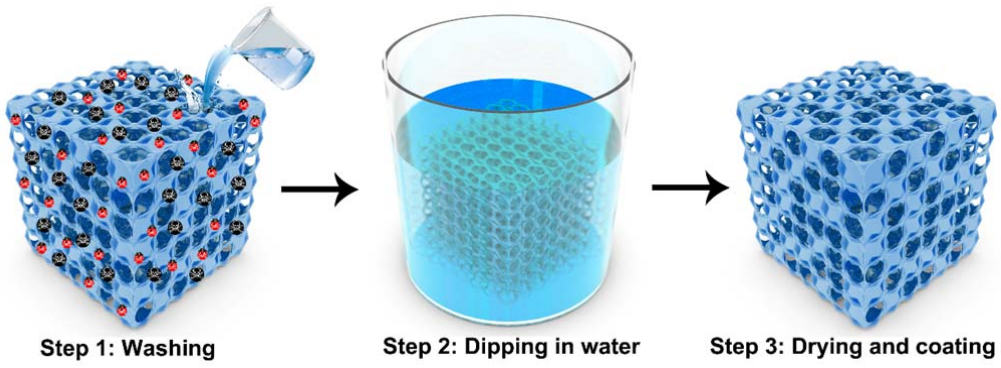

Supplementary **Figure 10 | Schematic illustration of the regeneration procedure for the dusty ILP@MF filter.**

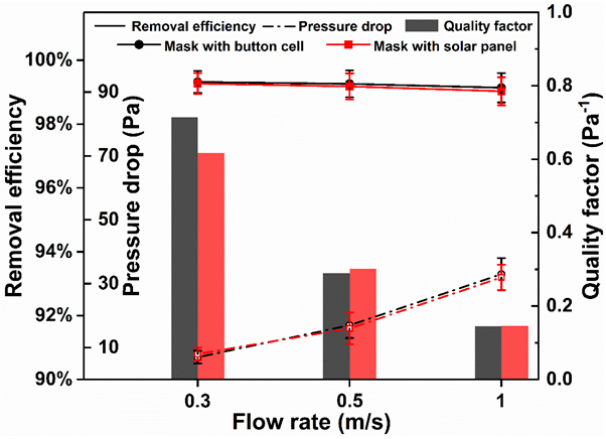

Supplementary **Figure 11 | Comprehensive index comparison including removal efficiency, pressure drop, and quality factor between masks made of button cell (black lines and pillars) and solar panel (red lines and pillars) under different flow rate.** Error bar represents the standard deviation of three replicate measurements. Source data are provided as a Source Data file.

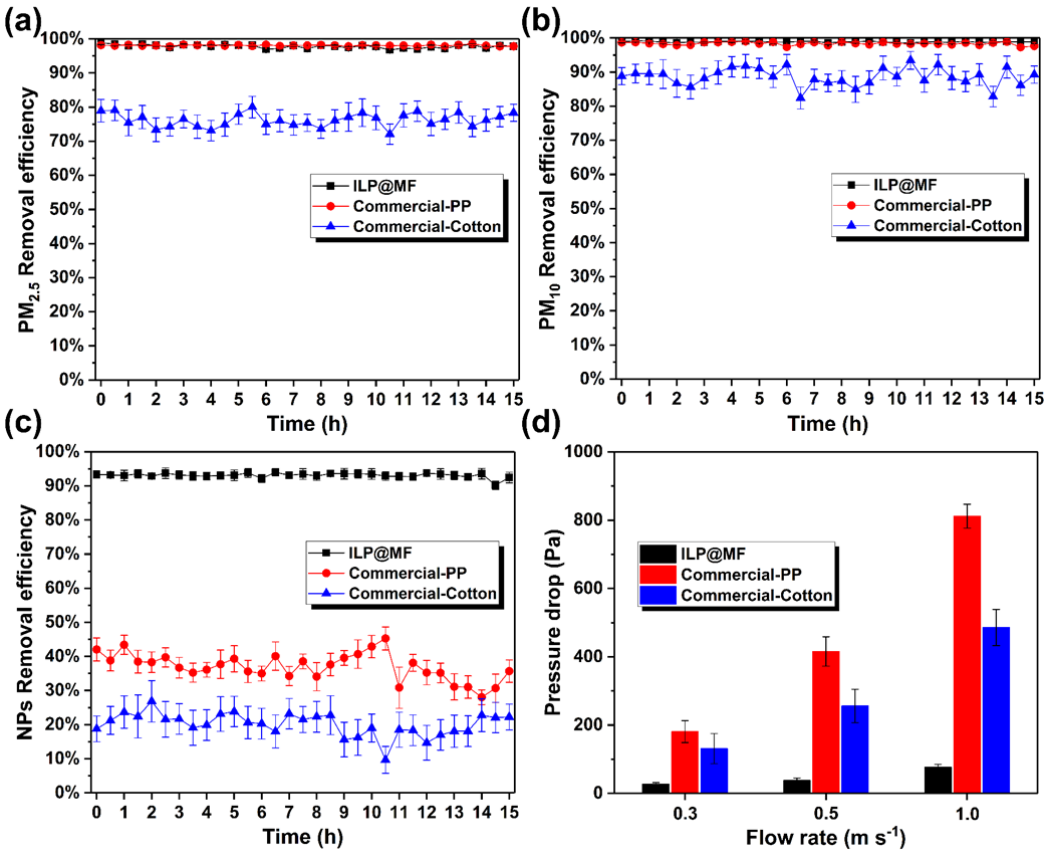

Supplementary **Figure 12 | Face masks filtration performance comparison between ILP@MF, commercial-PP, and commercial-cotton filters.** (**a**) removal efficiencies for $PM_{2.5}$. (**b**) removal efficiencies for PM_{10}. (**c**) removal efficiencies for NPs. (**d**) pressure drops in different flow rate conditions (0.3, 0.5, and 1 m s^{-1}). Error bar represents the standard deviation of three replicate measurements. Source data are provided as a Source Data file.

^{1}H NMR (400 MHz, DMSO-d_6)

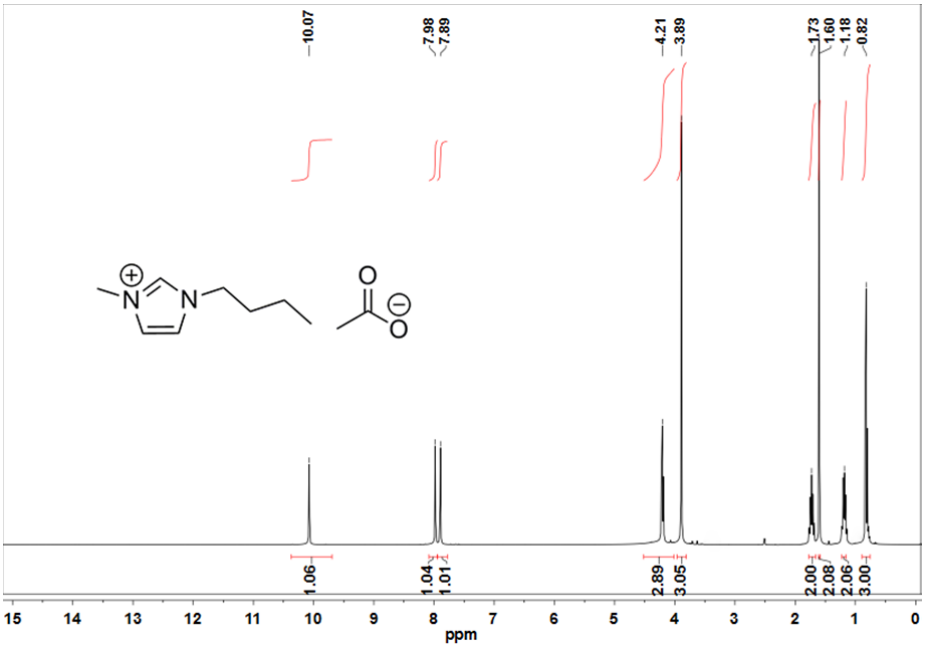

^{13}C NMR (101 MHz, DMSO-d_6)

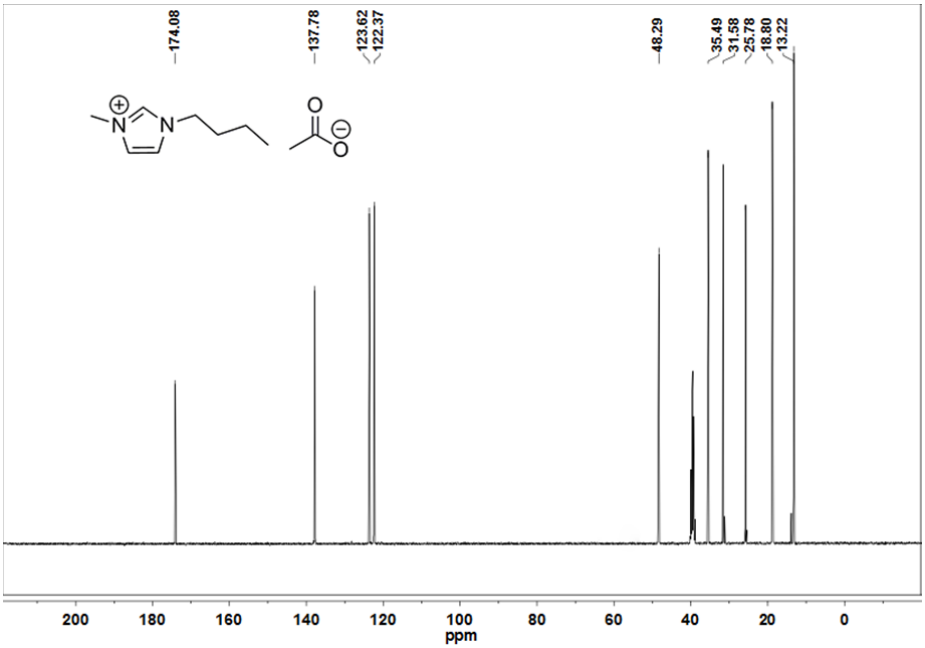

Supplementary **Figure 13 | ^{1}H and ^{13}C NMR spectra of [C_4mim][OAc].**

^{1}H NMR (400 MHz, DMSO-d_6)

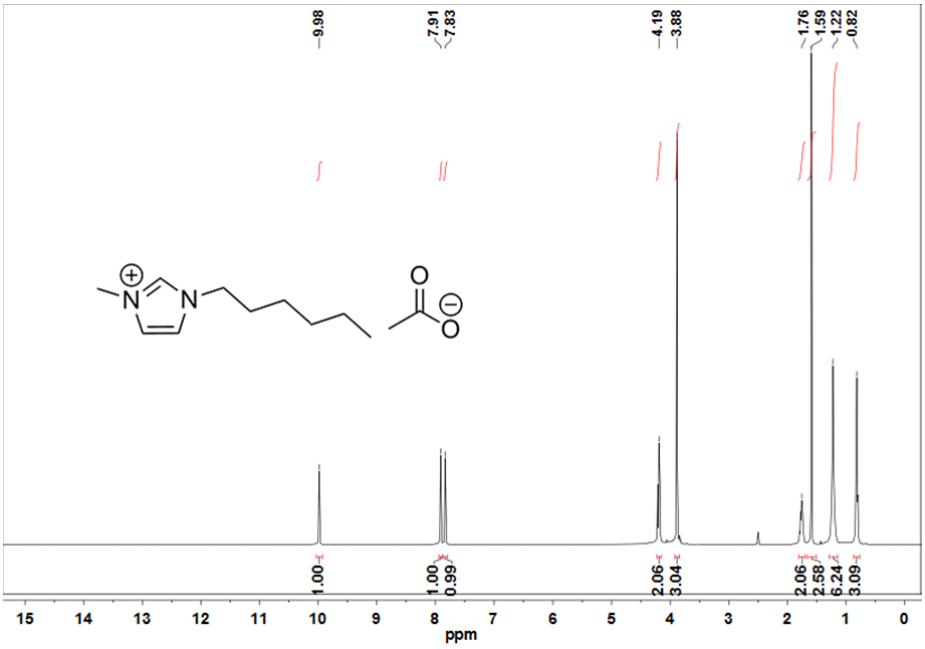

^{13}C NMR (101 MHz, DMSO-d_6)

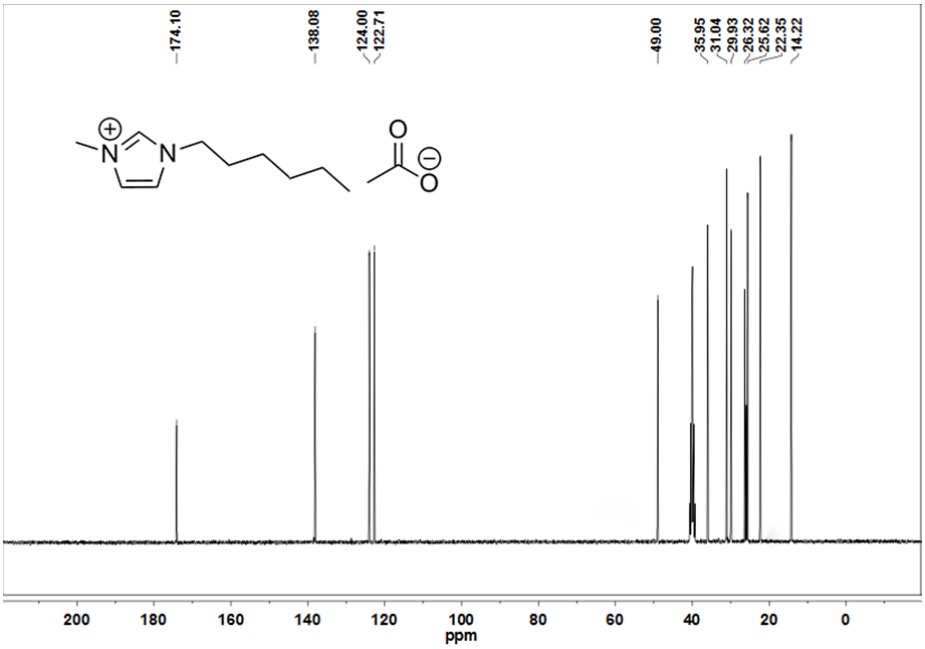

Supplementary **Figure 14 | ^{1}H and ^{13}C NMR spectra of [C_6mim][OAc].**

^{1}H NMR (400 MHz, DMSO-d_6)

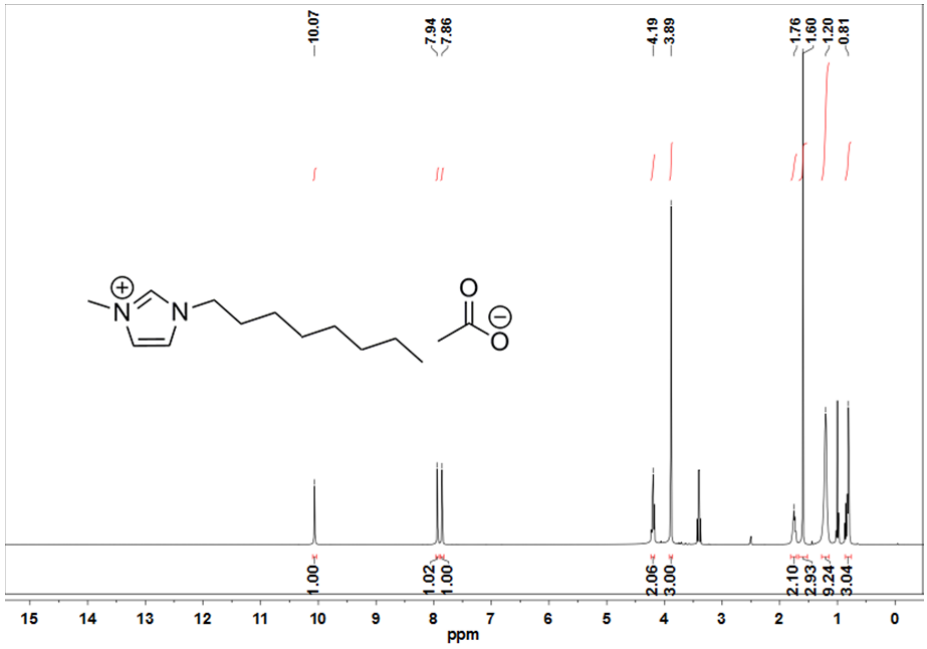

^{13}C NMR (101 MHz, DMSO-d_6)

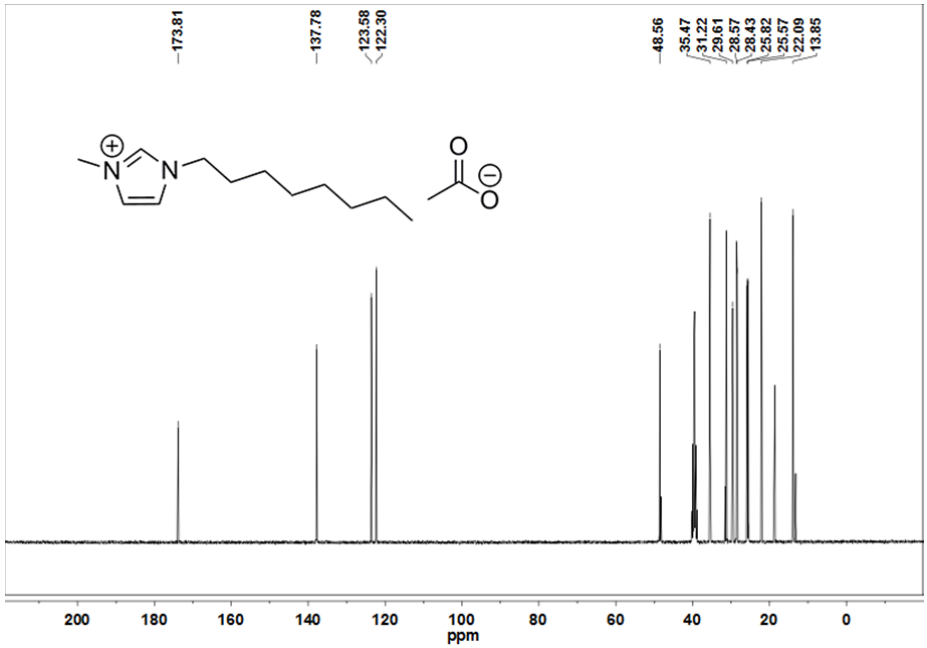

Supplementary **Figure 15 | ^{1}H and ^{13}C NMR spectra of [C_8mim][OAc].**

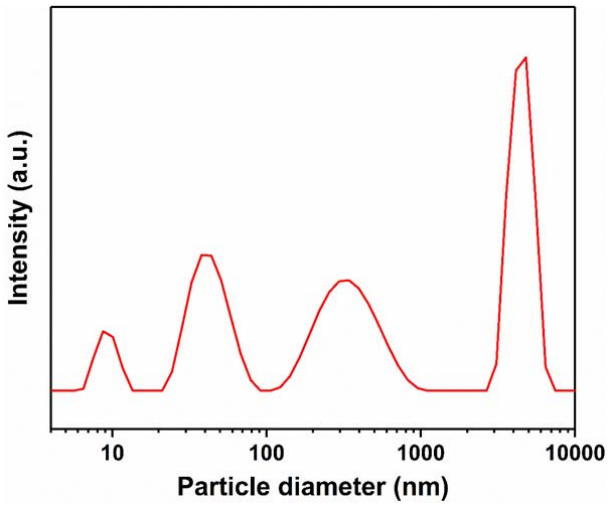

Supplementary **Figure 16 | DLS curves of the particles produced by the smoke of burned cigarette.** Source data are provided as a Source Data file.

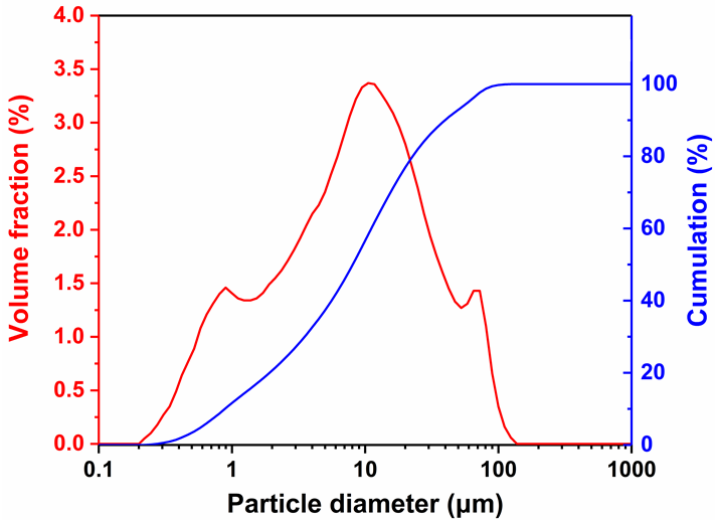

Supplementary **Figure 17 | Laser particle analysis curves of the particles produced by the smoke of burned cigarette.** Source data are provided as a Source Data file.

Supplementary **Table 2 |** The optimized geometry coordinates (Å) of the three simplified polymers including isobutyramide (simplified PAM), isopropanol (simplified PVA), and *N*-isopropyl pyrrolidone (simplified PVP).

Isobutyramide

C	-1.36089300	-1.26989900	-0.02423200
C	-0.61606100	0.00079000	-0.46292500
C	-1.36031600	1.27039000	-0.02013800
C	0.79303500	-0.00044400	0.13774000
N	1.82296800	0.00153900	-0.76170300
O	0.98790500	-0.00293000	1.34117500
H	-2.37459900	-1.27591000	-0.43386600
H	-0.85054800	-2.17419000	-0.36697600
H	-1.42560800	-1.30960800	1.06517300
H	-0.53846000	0.00256700	-1.55682100
H	-0.84948500	2.17557300	-0.35979700
H	-2.37397600	1.27827500	-0.42985900
H	-1.42520500	1.30656200	1.06938300
H	2.76892800	0.00095200	-0.41179600
H	1.67034200	0.00342300	-1.75558300

Isopropanol

C	-1.32872100	-0.55399400	-0.08970300
C	-0.00218600	0.03731800	0.36373500
C	1.20042900	-0.78088500	-0.10316100

145	O	0.04498600	1.37356400	-0.16268800
146	H	-1.46092500	-1.56111600	0.31442700
147	H	-2.15891100	0.06850200	0.25054800
148	H	-1.36579300	-0.60826800	-1.18132600
149	H	0.00617200	0.08734000	1.46264100
150	H	2.14015100	-0.32531400	0.22664200
151	H	1.16460300	-1.79512900	0.30560300
152	H	1.21652900	-0.84544800	-1.19477500
153	H	0.88114900	1.77628800	0.09251400
154				
155				
156	*N*-isopropyl pyrrolidone			
157				
158	C	-2.04697300	-0.21087300	-1.28706900
159	C	-1.41878000	-0.62328800	0.05187900
160	C	-2.13521300	0.00803000	1.25201000
161	N	0.02965400	-0.34928600	0.08119600
162	C	0.57840200	0.90115300	-0.02171300
163	C	2.09257400	0.73871400	-0.15189700
164	C	2.35533100	-0.72657700	0.22307100
165	C	1.01196500	-1.42224700	-0.06936000
166	O	-0.03416400	1.95680400	-0.03381100
167	H	-1.53975100	-0.69793100	-2.12464700
168	H	-3.10021100	-0.50376900	-1.31410000
169	H	-1.98349700	0.86995700	-1.42262600
170	H	-1.49968500	-1.71103300	0.14575000
171	H	-1.68893900	-0.32963900	2.19099700
172	H	-3.18920700	-0.28380000	1.25030600
173	H	-2.07259200	1.09560300	1.20953700
174	H	2.35972900	0.95575900	-1.19185500

H	2.60797500	1.46645800	0.47518400
H	2.58221000	-0.80577200	1.28920400
H	3.18075400	-1.17898000	-0.32833300
H	0.98394100	-1.83803600	-1.08680100
H	0.80118000	-2.23771700	0.62797200

Supplementary **Table 3 |** The optimized geometry coordinates (Å) of [C_4mim][OAc].

[C_4mim][OAc]

C	-0.22401600	3.08930100	0.13255200
C	0.95401300	2.55712500	-0.29116300
C	-0.54672200	0.91848500	-0.19882700
N	-1.14209200	2.05369200	0.18457900
H	-0.48461600	4.10076400	0.39288400
H	1.91045400	3.01836900	-0.46695000
H	-0.98466800	-0.12419200	-0.26892800
N	0.72903000	1.20603100	-0.49425200
C	-2.55468700	2.15158900	0.58371300
H	-2.61317800	2.56435200	1.59172700
H	-3.08167300	2.80386200	-0.11428300
H	-2.97385200	1.13862400	0.55793900
C	1.70986800	0.18896700	-0.91232100
H	2.26170300	0.59041500	-1.76631200
H	1.12894000	-0.67023700	-1.25143200
C	2.65628600	-0.21564200	0.21972800
H	2.05779000	-0.57925400	1.06151400

H	3.20887100	0.66248500	0.57517900
C	3.64191900	-1.30330200	-0.22247600
H	4.22758900	-0.94133900	-1.07642700
H	3.07828500	-2.17180600	-0.58042400
C	4.58910900	-1.73538700	0.90025400
H	4.03239900	-2.13382500	1.75344000
H	5.27524300	-2.51441800	0.55871500
H	5.19197700	-0.89498100	1.25835900
C	-2.54268100	-1.81722400	-0.03407900
O	-3.24496500	-0.86037600	0.36969000
O	-1.33538900	-1.72116100	-0.43086800
C	-3.15529400	-3.21855500	-0.06504300
H	-3.13748100	-3.60193600	-1.08921200
H	-2.54977500	-3.89717200	0.54222000
H	-4.18051300	-3.20762900	0.30510100

Supplementary **Table 4 |** The optimized geometry coordinates (Å) of three ILP composites including [C_4mim][OAc]-PAM, [C_4mim][OAc]-PVA and [C_4mim][OAc]-PVP.

[C_4mim][OAc]-PAM

C	-0.56524500	-2.27806400	-1.23675400
C	0.19999900	-1.24538300	-0.79013400
C	-1.92371200	-0.80910700	-0.27698400
N	-1.88063800	-1.98751600	-0.90851500
H	-0.29160100	-3.18571800	-1.74657000
H	1.26350100	-1.06915200	-0.82168000

233	H	-2.82265600	-0.27316200	0.13814400
234	N	-0.66855800	-0.34582700	-0.19405700
235	C	-3.05899600	-2.82057100	-1.18649100
236	H	-3.17231300	-2.93540700	-2.26579500
237	H	-2.92306600	-3.79970500	-0.72451400
238	H	-3.93178800	-2.31311300	-0.75811000
239	C	-0.31692400	0.95574300	0.39813700
240	H	0.60180600	0.81608300	0.97104600
241	H	-1.12682700	1.21184100	1.08310300
242	C	-0.13763400	2.04685200	-0.65952900
243	H	-1.06492600	2.13464400	-1.23561100
244	H	0.65324000	1.74808100	-1.35646700
245	C	0.20892800	3.40046500	-0.02960500
246	H	1.12694400	3.30169900	0.56248800
247	H	-0.58299200	3.68461300	0.67229300
248	C	0.38828400	4.50904600	-1.07082000
249	H	-0.52635400	4.65377200	-1.65316800
250	H	0.63372000	5.46266800	-0.59600000
251	H	1.19366300	4.26833400	-1.77216800
252	C	-5.06220200	0.05575100	0.77059900
253	O	-5.19147800	-1.05656700	0.20458800
254	O	-3.97344200	0.70569500	0.87283000
255	C	-6.29756900	0.69992600	1.40702700
256	H	-6.15388800	0.76201600	2.49002100
257	H	-6.41412000	1.72278700	1.03908100
258	H	-7.19714000	0.12188000	1.19394000
259	C	4.51413100	-0.66078100	2.27190100
260	C	5.13216300	-0.83227600	0.87535400
261	C	5.09485500	-2.29993900	0.41805100
262	C	4.37967800	0.03632800	-0.13304200

263	N	5.12025200	0.97059300	-0.78897900
264	O	3.18039800	-0.09767300	-0.34523000
265	H	5.04471800	-1.28218000	2.99760600
266	H	4.56735500	0.37695400	2.61170200
267	H	3.46475000	-0.96252500	2.26158600
268	H	6.17754500	-0.50464400	0.91222200
269	H	5.55321400	-2.42836700	-0.56623500
270	H	5.63635200	-2.92947700	1.12856200
271	H	4.06283000	-2.65316400	0.36280700
272	H	4.66743000	1.56096600	-1.47039000
273	H	6.10483900	1.08994700	-0.62080800
274				
275				
276	$[C_4mim][OAc]$-PVA			
277				
278	C	1.26561200	0.64650900	0.03181400
279	C	0.36238500	1.58722500	0.42095300
280	C	-0.70838600	-0.35315200	0.25539700
281	N	0.57477200	-0.55151800	-0.06623300
282	H	2.32264500	0.72252500	-0.17716400
283	H	0.48522400	2.63880100	0.61480900
284	H	-1.55271800	-1.09817700	0.27771600
285	N	-0.85758800	0.94361700	0.55770900
286	C	1.13355800	-1.85592400	-0.45076500
287	H	1.59429900	-1.77048800	-1.43555500
288	H	1.88633600	-2.15415100	0.28022000
289	H	0.30717200	-2.57538200	-0.47099500
290	C	-2.15105500	1.54526800	0.92071000
291	H	-1.98636800	2.19014000	1.78810000
292	H	-2.79662900	0.71938100	1.22409300

293	C	-2.78381700	2.32375700	-0.23467200
294	H	-2.90857700	1.64566300	-1.08528900
295	H	-2.10672500	3.12322000	-0.55917900
296	C	-4.14110000	2.92074500	0.15451600
297	H	-4.01370500	3.58771300	1.01603800
298	H	-4.80526000	2.11421600	0.48421400
299	C	-4.80141600	3.68908500	-0.99346800
300	H	-4.97497200	3.03727300	-1.85467600
301	H	-5.76701100	4.10040300	-0.68863900
302	H	-4.17508400	4.52245600	-1.32709800
303	C	-2.67468600	-3.11856500	-0.00338300
304	O	-1.52606300	-3.47447200	-0.36023500
305	O	-2.99267400	-1.94699800	0.38017700
306	C	-3.80195600	-4.15456900	-0.01918500
307	H	-4.21226600	-4.26232500	0.98900000
308	H	-4.61484600	-3.80238000	-0.66048000
309	H	-3.44391900	-5.12097000	-0.37481500
310	C	5.38786400	0.42805700	1.37310200
311	C	5.71412300	0.72634000	-0.08220700
312	C	6.55928000	-0.36236700	-0.73748100
313	O	4.45033200	0.87710400	-0.76539300
314	H	6.30519100	0.35136900	1.96184400
315	H	4.77174000	1.22201100	1.80075000
316	H	4.84437000	-0.51711600	1.45802200
317	H	6.25252300	1.68245400	-0.13735700
318	H	6.76745900	-0.12462600	-1.78581600
319	H	7.52079700	-0.46483900	-0.22668000
320	H	6.04197600	-1.32496900	-0.69980600
321	H	4.62287500	1.03357500	-1.69996200
322				

[C_4mim][OAc]-PVP

C	-0.20787800	-0.73315700	-0.40461800
C	-1.17710100	-1.62933600	-0.73639500
C	-2.16987300	0.30883300	-0.29664800
N	-0.85175100	0.46457500	-0.13459000
H	0.86639100	-0.83111000	-0.34247900
H	-1.10639200	-2.66514100	-1.01987300
H	-2.98726400	1.06767800	-0.17635000
N	-2.38959000	-0.95991600	-0.66537700
C	-0.21692400	1.72863000	0.26510000
H	0.27692400	1.59234500	1.22799000
H	0.52273100	2.00762300	-0.48553100
H	-1.00561600	2.48506900	0.33584400
C	-3.73382200	-1.50853100	-0.90494100
H	-3.71185900	-2.04431300	-1.85789800
H	-4.39745200	-0.64839200	-1.01016500
C	-4.20790800	-2.42062700	0.22875400
H	-4.18449600	-1.85476000	1.16605000
H	-3.51234100	-3.26038200	0.34589700
C	-5.62387300	-2.95302100	-0.01911200
H	-5.64811600	-3.49818200	-0.97069400
H	-6.30992100	-2.10639300	-0.13131300
C	-6.11808600	-3.86724600	1.10549000
H	-6.13819200	-3.33855000	2.06304100
H	-7.12992600	-4.22842500	0.90470000
H	-5.46945100	-4.74129800	1.22116500
C	-4.02749400	3.13146500	0.11349100
O	-2.83104000	3.45888700	0.30108100

O	-4.43459400	1.95213100	-0.13710600
C	-5.10351300	4.22001200	0.17746900
H	-5.58023900	4.31504200	-0.80280700
H	-5.88289000	3.92873400	0.88682200
H	-4.67599500	5.18010100	0.46783500
C	4.45115500	1.92210500	1.32292500
C	5.18400500	1.39747700	0.08114100
C	4.60718000	1.95508400	-1.22525000
N	5.23712600	-0.07855500	0.06161700
C	4.14835600	-0.89341900	0.00207700
C	4.62198100	-2.33417500	0.17509500
C	6.14483500	-2.25877600	-0.00519100
C	6.47984700	-0.79838000	0.35095000
O	2.98424900	-0.53282800	-0.13999100
H	4.90322800	1.53261000	2.23909800
H	4.50704300	3.01323600	1.35699200
H	3.40024600	1.63046500	1.30282300
H	6.23025700	1.71105800	0.14739500
H	5.17642400	1.59526700	-2.08610700
H	4.65654100	3.04705200	-1.21634400
H	3.56666900	1.65428000	-1.34832200
H	4.33775900	-2.65798100	1.18240200
H	4.11528400	-2.99011800	-0.53331800
H	6.40825500	-2.44994400	-1.04823800
H	6.69384200	-2.96932000	0.61364500
H	6.75177200	-0.68848800	1.40955500
H	7.30205500	-0.39763800	-0.24725700

References

1. Owens, D. K. & Wendt, R. C. Estimation of the surface free energy of polymers. *J. Appl. Polym. Sci.* **13**, 1741-1747 (1969).
